# A Statistical Method of Identifying Interactions in Neuron–Glia Systems Based on Functional Multicell Ca2+ Imaging

**DOI:** 10.1371/journal.pcbi.1003949

**Published:** 2014-11-13

**Authors:** Ken Nakae, Yuji Ikegaya, Tomoe Ishikawa, Shigeyuki Oba, Hidetoshi Urakubo, Masanori Koyama, Shin Ishii

**Affiliations:** 1 Integrated Systems Biology Laboratory, Graduate School of Informatics, Kyoto University, Sakyo-ku, Kyoto, Japan; 2 Laboratory of Chemical Pharmacology, Graduate School of Pharmaceutical Sciences, The University of Tokyo, Bunkyo-ku, Tokyo, Japan; 3 Center for Information and Neural Networks, Suita City, Osaka, Japan; University of Connecticut, United States of America

## Abstract

Crosstalk between neurons and glia may constitute a significant part of information processing in the brain. We present a novel method of statistically identifying interactions in a neuron–glia network. We attempted to identify neuron–glia interactions from neuronal and glial activities via maximum-a-posteriori (MAP)-based parameter estimation by developing a generalized linear model (GLM) of a neuron–glia network. The interactions in our interest included functional connectivity and response functions. We evaluated the cross-validated likelihood of GLMs that resulted from the addition or removal of connections to confirm the existence of specific neuron-to-glia or glia-to-neuron connections. We only accepted addition or removal when the modification improved the cross-validated likelihood. We applied the method to a high-throughput, multicellular *in vitro* Ca2+ imaging dataset obtained from the CA3 region of a rat hippocampus, and then evaluated the reliability of connectivity estimates using a statistical test based on a surrogate method. Our findings based on the estimated connectivity were in good agreement with currently available physiological knowledge, suggesting our method can elucidate undiscovered functions of neuron–glia systems.

## Author Summary

Many neuroscientists believe that neurons mainly perform information processing in the brain. Glial cells have traditionally been regarded as passive cells, whose roles have been limited to mechanical support and energy transfer to neurons. However, some studies have recently demonstrated the existence of interactions between neurons and glial cells and implied the involvement of crosstalk between neuronal and glial systems in information processing. Nevertheless, the details on neuron–glia communication largely remain unknown. One way of addressing this issue is to use a powerful statistical methodology to identify the network structure based on high-throughput time-lapse imaging from neuron–glia networks. We developed a new statistical method for functional connectivity analysis that was suitable for examining neuron–glia interactions. We applied the method to multicellular Ca2+ imaging data, where neurons and glial cells carried out spontaneous activities in a rat hippocampal CA3 culture. We found in a data-driven manner that each glial cell facilitated the activities of neighboring neurons with a peak latency of 500 ms. Our study is the first of its kind to present a statistical framework to investigate the functional connectivity between neurons and glial cells. Our statistical method is thus capable of identifying neuron–glia interactions by utilizing the high-throughput imaging technique.

## Introduction

Information processing in the brain is primarily performed by neurons [1], [2]. Some studies, however, have revealed the existence of crosstalk between neurons and astrocytes [3]–[6], [6]–[14] that neighbor the neurons and envelop the neuronal synapses [15]. The observations in these studies suggest the involvement of glia in the brain's information processing [16]. Stimulation applied to the main type of glial cells (i.e., astrocytes) may induce the exocytosis of gliotransmitters, which in turn modulates post-synaptic currents [17] and increases post-synaptic excitability [18], [19]. Stimulation applied to neurons, on the other hand, elevates the Ca2+ activity of astrocytes [8]. This effect occurs both in culture and in acute brain slices, and is most likely mediated by astrocyte receptors for neuro-active molecules, neurotransmitters and neuromodulators [8]. *In vitro* astrocytes are known to exhibit relatively slow non-electrical activities (100 ms
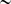
1 min) [15]. In contrast, neurons exhibit rapid depolarization, or ‘spikes’ (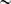
1 ms). Furthermore, *in vivo* animal experiments have suggested that glia affect neural networks in the sensory cortex [20], [21] and in the motor cortex [22]. These *in vivo* results imply that glia may play an important role in the information processing associated with sensory and motor functions. These findings clarify the necessity to shift our focus from pure neuronal networks to neuron–glia networks [23]–[26]. Unless otherwise noted, we will denote astrocytes as glia after this.

To clarify the roles of neuron–glia interactions in brain information processing, we need to examine neuronal and glial activities in a network in an unmanipulated state. For example, some experiments have artificially generated epileptiform bursting activities of neurons and glial cells, and then examined the contributions of glial activity via further pharmacological manipulation [6], [7], [27]. Such approaches are very appropriate for clinical applications. However, one needs to assess the concise contribution of glial activities in networks in a resting state to elucidate their functions in information processing. In this case, the sheer complexity of the networks makes it extremely difficult to estimate neuron–glia interactions. The dissociation of glial effects from other neuronal effects is a challenging problem, especially when indirect interactions via other neurons in the network are taken into consideration. Also, such indirect interactions may themselves be important for identifying neuron–glia interactions.

Generalized linear models (GLMs) have been developed for pure neuronal networks (without glia) to analyze their interactions in terms of both response functions and functional connectivity [28]–[33]. One can identify the characteristics of multivariate time series by estimating the model parameters in the GLM-based approaches. In the framework of the GLMs, the probability of spike events in a network at any given time depends on the history of the activity time series. The response functions and functional connectivity are estimated from the observed time-series of multi-neuronal spiking activities. The estimated response functions measure the extent to which the other neuronal spikes causally affect the spiking activities of target neurons. The estimated functional connectivity, on the other hand, represents the pathways over which the neuronal activities propagate. Although the functional connectivity does not necessarily correspond to a specific synaptic or non-synaptic connection (e.g., gap-junction) [34], [35], existing studies have shown that synaptic connections are closely linked to the connections that can be functionally estimated based on Ca2+ imaging [36] and multi-electrode physiological measurements *in vivo* [37], [38]. Friston et al. argued that functional connectivities, particularly the ones that depend on the context of environments and behaviors, represent information flow propagating through anatomical connectivity [39] in their research on fMRI datasets. One may then use the response functions and functional connectivity to address how each component contributes to information processing in the brain, either in a controlled environment or in the resting state. This type of data-driven approach is important in analyzing experimental data with high throughput, and in our particular case of identifying unknown neuron–glia interactions, even with a lack of a priori biological knowledge.

Neuronal spiking activity is binary, while glial activity may be regarded as being graded time series [18]. Since we cannot directly apply the existing GLM-based techniques to such heterogeneous neuron-glia networks, we propose a new GLM-based statistical method in this paper to identify the interactions between neurons and glial cells. We applied this statistical method to the time-lapse imaging data of the rat hippocampal CA3 region based on high-resolution (184
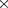
94 pixels) and high-speed (100 Hz) Ca2+ imaging [40]. We determined the response functions and functional connectivity of the neuron–glia network from spontaneous activities of neurons and glial cells, which were then quantified by measuring the Ca2+ signal averaged over each cell. The reliability of the determined connectivity was evaluated with a statistical test based on a surrogate method. Our analysis revealed several characteristics of interactions between neurons and glia, including the positive effect of glial activities on the activities of neighboring neurons. These results obtained solely by using the proposed method were compatible with existing knowledge on neuron–glia interactions, reinforcing the previous neurobiological observations and providing new insights into the functions of neuro–glia systems.

## Results

### Methods overview

**Figure 1:**
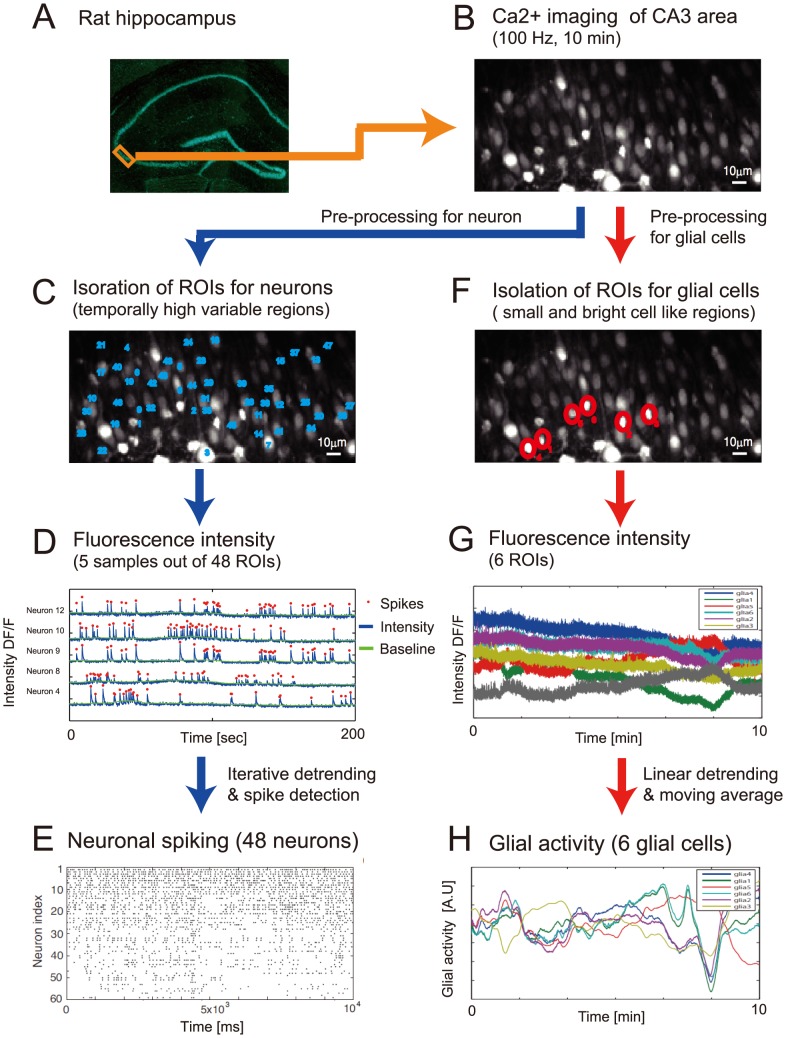
Outline of image preprocessing. (A) The rectangle indicates the target circuit of our analysis, a part of the hippocampal CA3 region of a rat, whose area was 184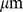 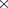
94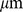. (B) The average Ca2+ fluorescence image over the whole observation period of 10 min. (C) Neuronal ROIs were defined as the regions exhibiting sufficiently large temporal variance within the Ca2+ imaging data (blue numerals. For more details on the detection procedure, see Methods). (D) Neuronal spikes in each ROI were detected as signal peaks (red points) with substantially high intensities in comparison to the standard deviation within the baseline. The baseline was estimated with an iterative procedure (see Methods). The blue line indicates the signal profile after baseline correction that includes detrending. (E) A spike profile for the ROIs from which we selected 48 ROIs that showed high frequencies of spikes. (F) We selected small and bright cell-like regions as glial ROIs (for more details, see Methods) in parallel with the detection of neuronal ROIs. (G) We took the time series as the average signal intensity within the ROI region for each glial ROI. (H) We obtained the activity time series of six glial ROIs after linear detrending and smoothing.

We developed a statistical method to identify the functional connectivity and response functions of neuron–glia networks *in situ*, which may reflect the dynamics of ionic receptors on neurons and glial cells. We applied it to a Ca2+ imaging dataset of an *in vitro* brain slice (see ‘ *In vitro* Ca2+ imaging’ section in Methods), by using the Ca2+ signal (concentration) as an indicator of neuronal as well as glial activities. We conducted high-resolution (184
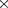
94 pixels) and high-speed Ca2+ imaging (100 Hz) from a CA3 region (184 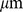 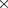
94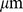) of a rat's hippocampal slice to prepare the dataset by using Nipkow-type spinning-disk microscopy [40]. We observed spontaneous Ca2+ activities of neurons and glial cells within the 10 min of a fluorescence image series. An image preprocess applied to the image series extracted binary activities of 48 neurons and graded activities of six glial cells (Figs. 1E and 1H). The spike frequency of the 48 neurons was 0.03–1 Hz. The activity dataset thus consisted of the observation time series of 48 neurons and six glial cells.

**Figure 2:**
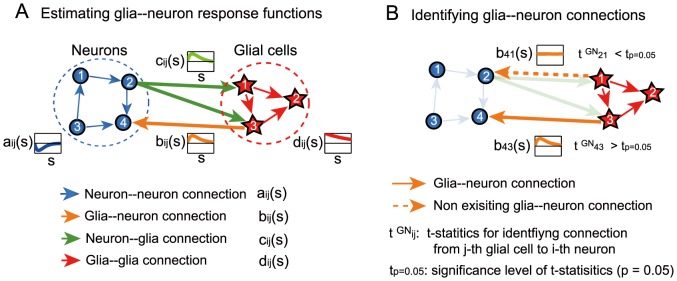
Outline of functional connectivity analysis. (A) We statistically estimated the whole network of neurons and glial cells based on the neuronal and glial activities obtained from time-lapse Ca2+ imaging. A neuron–glia system consists of four types of possible connections (depicted by arrows): between neurons (blue), from glial cells to neurons (orange), from neurons to glial cells (green), and between glial cells (red). (B) Each specific connection in the neuron-glia network was identified by basically comparing the cross-validated likelihood between two network structures: (1) one with the connection and (2) the other without the connection.

We tried to identify the neuron–glia system based on this observation time series by estimating the parameters of our neuron–glia network model (Fig. 2A. See ‘Generative model and MAP estimation’ section of Methods). We developed a generalized linear model (GLM) of a neuron–glia network as a variation of previous GLMs used for neuronal networks [41]. We could efficiently and uniquely obtain maximum a posteriori (MAP) estimates of the parameters by assuming that the present activities of neurons and glial cells were independent conditional on their past. Using the MAP estimates, we could avoid ‘overfitting’, where the model estimates were disturbed by noise involved in the relatively short observation time series.

We evaluated the quality-of-fit of the estimated model to the observation time series by using 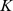-fold cross-validation (see ‘Functional connectivity analysis’ section of Methods). The observation time-series dataset in the 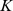-fold cross-validation was partitioned into 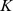 subseries. A single subseries was used as the dataset to evaluate the estimated model, while the remaining 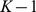 subseries were used as the training dataset to estimate the model parameters. Our measure of the quality-of-fit was the cross-validated likelihood, i.e., the model's predictability of the activities of neurons and glial cells in the test dataset averaged over 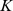 folds (for more details, see ‘Functional connectivity analysis’ in Methods section).

Since the cross-validated likelihood depended on the network structure of the model, i.e., the connectivity pattern within the neuron–glia system, it could be used to identify the connectivity between neurons and glial cells. For a specific connection from a glial cell to a neuron (a glia-to-neuron connection), we accepted the connection if a network structure with the new connection indicated a better cross-validated likelihood than the network structure that did not include the connection. In contrast, for a specific connection from a neuron to a glial cell (a neuron-to-glia connection), we preferred a network structure without the connection if the reduced network structure indicated a better cross-validated likelihood than the one with the connection (see ‘Functional connectivity analysis’ section in Methods). We identified the best network structure, i.e., the connectivity and response functions of the neuron–glia system, by repeating this set of procedures (the addition/removal of connections including MAP-based parameter estimation inside). The reason for our different treatment of glia-to-neuron and neuron-to-glia connections will be discussed later.

We conducted surrogate analysis to verify the reliability of the extracted functional connections as follows. First, we created a set of artificial time series for neurons and glial cells by applying “cyclic” rotations in which the cross correlations were destroyed but the autocorrelations were preserved. We then applied our algorithm to this artificial data set, and compared the number of identified connections against the number of connections we had identified from the original data. This obtained a statistical evaluation of the bulk number of connections that could be identified with our method.

Recent studies have shown that glial activities affect neuronal activities on various time scales, ranging from several tens of milliseconds to several hours [6], [12], [14]. We focused on interactions that lasted for a relatively short duration with a delay ranging between 100 and 500 ms in this study. This is because our method could not deal with interactions with longer delays in our time-lapse image dataset of 10 min (see ‘Limitations of proposed method’ in the Discussion section). A detailed description of the overall method is found in Methods and Text S1. The codes for our generative model and statistical analyses have been uploaded to GitHub (https://github.com/nakae-k/glia-neuron).

### Response functions of neuron–glia interactions

We estimated the response functions, 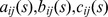, and 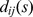, which corresponded to the connections between neurons, the connections from glial cells to neurons, the connections from neurons to glial cells, and the connections between glial cells (see Fig. 2A and ‘Generative model and MAP estimation’ in Methods). Here, 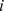 denotes the index of the “sender” cells, 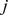 denotes that of the “receiver” cells, and 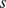 denotes the delay time.

**Figure 3:**
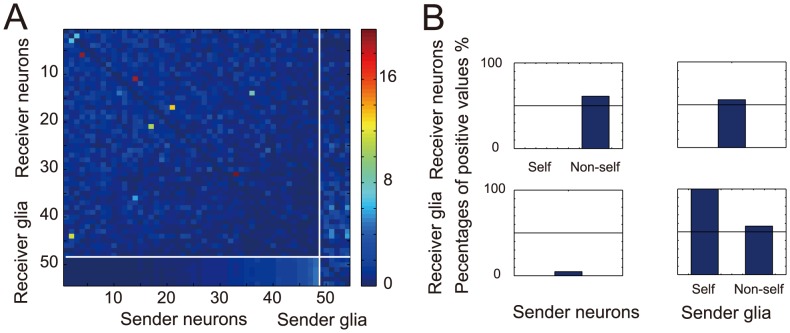
Neuron–glia network estimated from Ca2+ imaging data. (A) Connectivity matrix of the neuron–glia network estimated with our method. Each column and each row of the matrix correspond to “sender” (i.e., from) neuron/glia and “receiver” (i.e., to) neuron/glia. Indices of 48 neurons and indices of six glial cells are segmented by white lines on the matrix. Each matrix entry denotes the root mean square of the corresponding response function; the root mean square is normalized within the entry values of 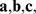 and 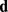 individually. This is because the magnitude of the response functions was considerably different across 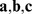 and 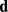. For example, the element (1, 49) indicates the magnitude of the response function from neuron 1 to glial cell 1 (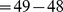). (B) The proportions (as percentages) of the response functions, 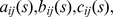 and 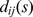, which took positive values are depicted in the top left, top right, bottom left, and the bottom right panels, respectively. The self-feedback connections of neurons, represented by 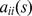, were all inhibitory, which would demonstrate the refractoriness of neurons. On the other hand, the self-feedback connections of glial cells, represented by 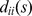, were all excitatory. This could be due to the timescale of the glial activities, which are much slower than the sampling frequency.

Fig. 3A shows the identified connectivity matrix of the neuron–glia network. Here, we assumed that the functional connections between neurons and glia were directional because the neuron-to-glia and the glia-to-neuron connections are believed to depend on different biophysical processes [23]. There are small numbers of connections with substantially larger values than the other connections at the top left of the matrix, i.e., inter-neuronal connections. This observation is consistent with existing physiological studies, which report that the strength of inter-neuronal connections in the hippocampus obeys a log-normal distribution [42] We can also see some strong glia-to-neuron connections at the top right.

We took temporal averages of 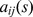 and 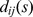, and determined connections corresponding to positive values as excitatory. We similarly determined connections corresponding to negative values as inhibitory. Approximately half of the inter-neuronal connections were found to be excitatory (Fig. 3B). This may suggest some sort of a balance in inter-neuronal and inter-glial connections. Positive values for the temporal averages of 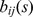 and 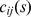 were found for 63% of the former and for 11% of the latter, suggesting that there were major excitatory effects from glial cells to neurons but minor inhibitory effects from neurons to glial cells.

### Functional connectivity analysis between neurons and glia

**Figure 4:**
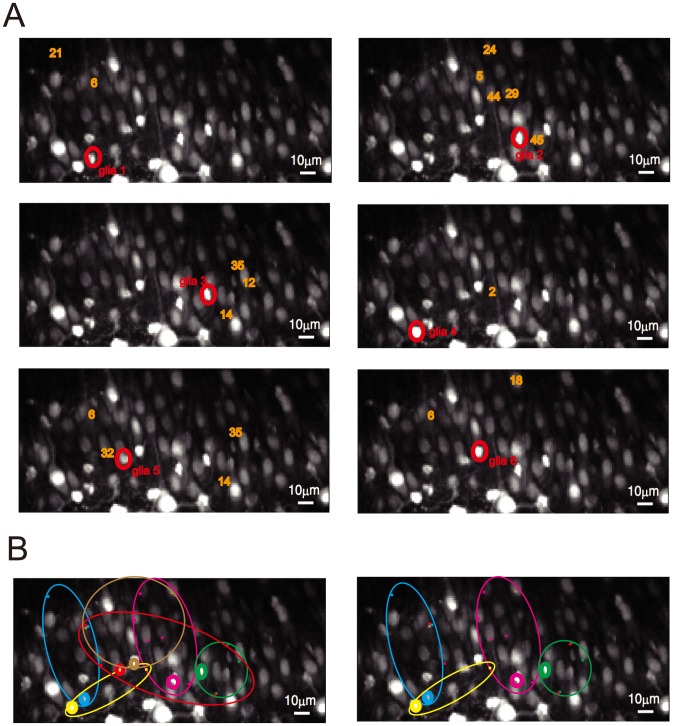
Identification of glia-to-neuron connections. (A) Connections from glial cells 1, 2, 3, 4, 5, and 6 to the 48 neurons, all of which were identified using the *t*-statistics, 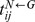, are shown in the top left, top right, middle left, middle right, bottom right and bottom left panels, respectively. Each ROI labeled by an orange numeral indicates the neuron that gave the better cross-validated likelihood if the network structure included the corresponding glia-to-neuron connection. (B) Visualization of projection range of each glial cell. (Left) Projection ranges of the six glial cells are visualized. The color of each ellipse corresponds to that of the “sender” glial cell. (Right) Projection ranges of four glial cells out of the six to enable better visibility.

We determined the existence of a connection (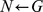) from the *j*-th glial cell to the *i*-th neuron using a newly designed *t*-statistic, 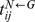, which determined whether the increase in the cross-validated likelihood resulting from the addition of the new connection was significant or not (see ‘Functional connectivity analysis’ section in Methods). We found that 24% of the glia-to-neuron pairs increased the cross-validated likelihood, and the remaining 76% decreased the cross-validated likelihood (Fig. S3). We also found that only 17 out of 288 possible glia-to-neuron connections could significantly increase the cross-validated likelihood (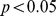) by performing the statistical test based on 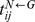. This suggested sparsity in glia-to-neuron connections (Fig. 4A). When we compared the activities of a neuron–glia pair that was identified as connected (e.g., neuron 6 and glial cell 2) with another pair that was identified as not connected (e.g., neuron 6 and glial cell 1), the correlation between the neuronal firing rate and glial activity was higher for the connected pair (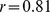) than that for the non-connected pair (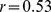) (Fig. S4).

**Figure 5:**
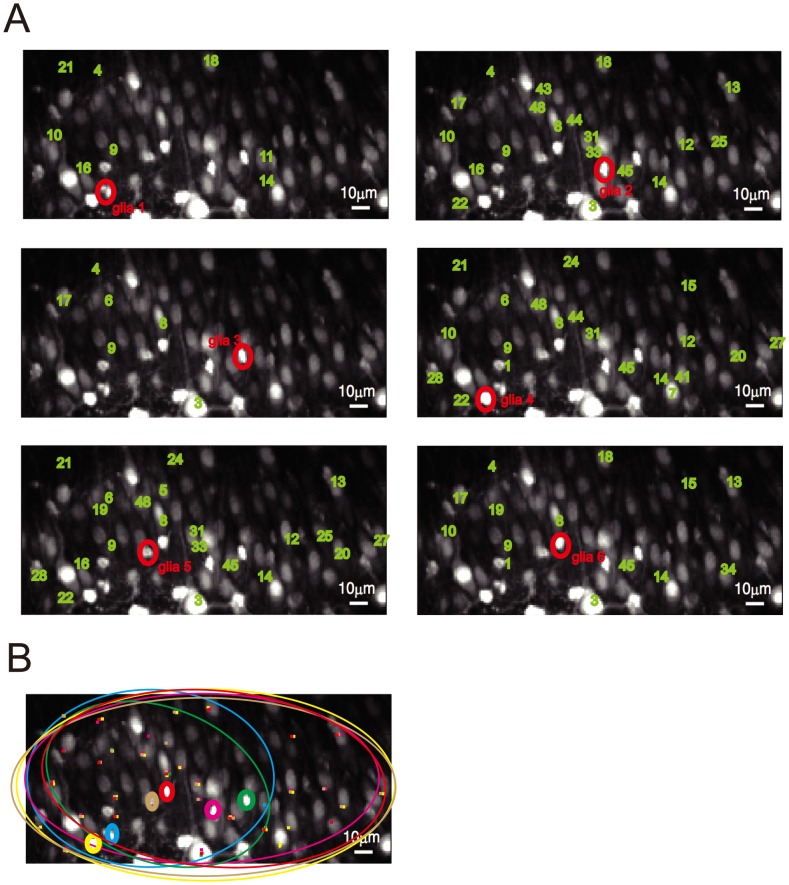
Identification of neuron-to-glia connections. (A) Connections from the 48 neurons to glial cells 1, 2, 3, 4, 5, and 6, all of which were identified using the *t*-statistics, 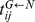, are shown in the top left, top right, middle left, middle right, bottom right and bottom left panels, respectively. Each ROI labeled by a green numeral indicates a glial cell for which the model's cross-validated likelihood deteriorated when the corresponding neuron-to-glia connection was removed. (B) Visualization of projection range to each of the six glial cells. The color of each ellipse corresponds to that of the “receiver” glial cell.

We also identified 89 neuron-to-glia connections out of 288 neuron-to-glia pairs with a similar *t*-statistic, 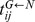 (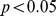), where 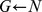 denotes the neuron-to-glia connection (Fig. 5A) (see ‘Functional connectivity analysis’ section in Methods). The average response function of the identified neuron-to-glia connections suggested small and inhibitory effects of neuronal activities on glial activities. The *t*-test (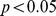) determined the temporal average of the response functions to be significantly negative. These results seemed to be inconsistent with those in experimental studies [8], [27], which have demonstrated excitatory neuron-to-glia connections. This inconsistency can be attributed to effects from other brain areas that were not considered in our study (e.g., the dentate gyrus), or to different experimental conditions. We need to emphasize that we observed spontaneous activities in our experiment while the preceding experiments mostly measured activities evoked by stimulation [19], [43] (also see Discussion).

We examined the reliability of connectivity from each of the six glial cells to neurons, measured in terms of the bulk number of identified connections by using the surrogate method (see ‘Surrogate method’ in Methods). We prepared 1000 surrogate glial activities for each glial cell. This analysis suggested that glial cells 2 and 5 had significantly large numbers of connections to neurons (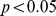). We similarly examined the reliability of connectivity from neurons to each of the six glial cells, measured in terms of the bulk number of identified connections. This analysis indicated that no glial cells received a significantly large numbers of connections from neurons (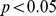).

### Spatial and temporal features of identified connections

The identified 17 glia-to-neuron connections out of 288 glia-to-neuron pairs are depicted in Fig. 4A. These connections had an interesting topological character, i.e., the range of functional connectivity from glia to neurons was local (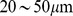, see Fig. S6). We performed the following statistical test to statistically confirm this observation. We let 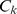 be the set of identified connections from the *k*-th glial cell to the 48 neurons and let 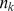 be the size of 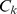. The values of *n_k_*’s were 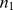 = 2, 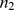 = 5, 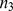 = 3, 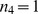, 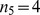 and 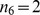. We then randomly selected 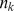 neurons from the total of 48 neurons for each glial cell 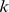, and measured the distance between the *k*-th glial cell and all the selected neurons. We then computed the median distance of such random glia-to-neuron connections over the six glial cells. We repeated this sampling 1000 times to obtain an empirical distribution of the median distance of randomly prepared glia-to-neuron connections. When the median distance of the glia-connected neurons from their respective glial cells was compared against this empirical distribution, it was found to be significantly lower (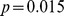).

**Figure 6:**
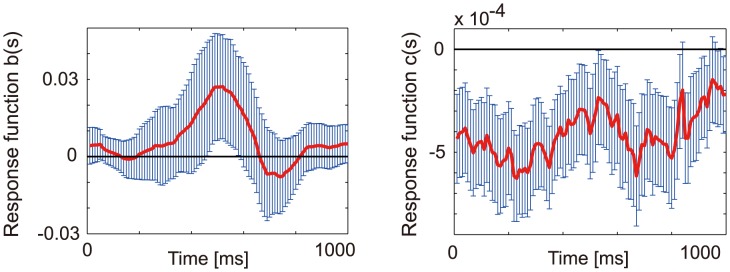
Response functions from neurons to glial cells and from glial cells to neurons. (Left) The estimated response functions of the identified connections from glial cells to neurons; the average and the 95% confidence intervals of the response functions, 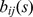, are plotted by the red curve and blue intervals, respectively. (Right) The estimated response functions of the identified connections from neurons to glia; the average and the 95% confidence intervals of the response functions, 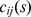, are plotted by the red curve and blue intervals, respectively.

We found from visual inspections that each neuron had some tendency to be under the functional projection of a unique glial cell. This tendency was particularly strong for neurons under the functional projection of glial cells 1, 2, 3, and 4 (Fig. 4B). These findings are consistent with the anatomy of astrocytes, where they are known to occupy nonoverlapping local territories whose diameter is about 30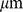. The findings are also in agreement with the hypothesis of functional islands of neurons modulated by individual astrocytes [44], [45]. Fig. 6 (left) suggests that the excitatory glia-to-neuron connections have a mean peak latency of around 500 ms. The *t*-test (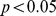) determined the temporal average of the response functions to be significantly positive.

The 89 neuron-to-glia connections identified from 288 neuron-to-glia pairs, on the other hand, were found to be non-local (Fig. 5B). When we actually applied a statistical test similar to that above to the identified neuron-to-glia connections, the *p*-value was 0.385 (also see Fig. S6). The average response function of the identified neuron-to-glia connections suggests small and inhibitory effects of neuronal activities on glial activities. The *t*-test (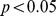) determined the temporal average of the response functions to be significantly negative.

## Discussion

### Identified connectivity and response functions

Our results suggested the existence of functional connectivity from glial cells to neighboring neurons within a 20 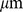 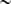
50 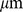 perimeter. The identified functional connectivity also exhibited a distinctive local tiling pattern with few overlaps (Fig. 4). Further, these connections had positive response functions on the time scale of 500 ms. These results are in good agreement with experimental findings [6], [44], [46]. For example, the activation of hippocampal CA1 astrocytes has induced an inward current to neurons for a duration of 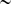
500 ms (e.g., [6]), which is mediated by glutamate released from astrocytes [19]; this phenomenon synchronizes the activities of CA1 neurons in the same range of 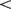
100 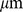 [47]. Anatomical studies have also found that astrocytes in the hippocampus occupy non-overlapping domains [44], [46]. The identified response functions correspond to the inward current to neurons, and the identified local connectivity corresponds to the mostly non-overlapping domain of astrocytes. This would also suggest that glial activities could affect neuronal information processing in spontaneously active situations, in concert with inter-neuronal and inter-glial interactions, like those in our *in vitro* experiment.

The estimated glia-to-neuron response functions had a time scale of several hundred milliseconds with a peak latency of 500 ms. This relatively long duration might include the time for the activations of neuronal AMPAR and NMDAR in response to gliotransmitter release. Because the deactivation kinetics of AMAPR is known to be very rapid (∼5 ms), one may think that AMPAR activation should not appear in the response functions derived from the sampling interval of 10 ms. However, response functions not only depend on receptor kinetics, but also on the entire processes of AMPAR-mediated transmission (i.e., from glial vesicle release to neuronal Ca2+ signals). These entire processes are known to require at least several hundred milliseconds [48]. Thus, the effects of AMPA- and NMDAR-mediated transmission were most likely reflected in our response functions.

Our analysis indicated the possible presence of many neuron-to-glia connections. We also found that, even if these connections really existed, the intensities of these connections were weak and they were spatially unlocalized. Indeed, neuron-to-glia interactions has been discovered in previous studies [8], [9], [49]. Although this has been observed in the bursting state of neuronal activities, such neuron-to-glia interactions may have been too small to observe in our spontaneously active situation. Thus, the identified weak neuron-to-glia connections were insignificant with a short observation time of 10 min. In contrast, if there were in fact no neuron-to-glia connections, those misidentified neuron-to-glia connections may have been due to spurious correlations between neuronal and glial activities. Such correlated activities may have been mediated by dentate gyrus (DG) neurons. DG neurons are known to relay signals to both CA3 astrocytes and CA3 neurons [50], [51]. Thus, CA3 astrocytes and CA3 neurons could have simultaneously responded to DG neurons, which might have resulted in correlated activities for the misidentified functional connections. In either case, the significantly longer and simultaneous observation of both CA3 and DG regions is necessary to address the origin of the identified weak and spatially unlocalized neuron-to-glia connections.

Ca2+ signal has been recognized to be one of the most powerful indicators of glial activities. For example, the transmission of gliotransmitter, glutamate, is known to depend on the glial Ca2+ concentration [52]. When a glial cell uptakes glutamate spilled out from synaptic clefts, the intracellular Ca2+ concentration of the glial cell is known to increase [8], [49], [53]. Although Ca2+ imaging is no doubt a powerful experimental methodology, our statistical method has potential applications to other types of imaging experiments. For example, we may apply our statistical technique to the dataset from intracellular pH imaging. Intracellular pH is known to reflect gliotransmitter release, which is a type of glial activity [54], [55].

When our method is applied to electrophysiological or imaging experiments from different hippocampal areas such as CA1, CA3, and the entorhinal cortex, it should be modified by, for example, changing the tuning parameters in the estimation (see ‘Tuning parameters’ section in Methods). Indeed, we should consider the possibility that the neuron–glia interactions are characterized by different biophysics in different brain regions [19], [56] and hence are represented by different tuning parameter values in our method.

Fig. 3B shows that about half the inter-neuronal and inter-glial interactions were positive and half were negative (i.e., the excitatory and inhibitory effects were balanced). The balanced excitatory and inhibitory effects in inter-neuronal interactions are known to lead to high levels of variability in neuronal spiking and this high variability can enable neuronal networks to embed rich information into their activity patterns [57], [58]. Our results suggest that this balance was not only achieved in inter-neuronal interactions but also in inter-glial interactions. Balanced inputs from the glial cells might similarly provide high levels of variability to glial activities and promote efficient information processing.

### Comparison with other approaches

Our method of identifying the functional connectivity between neurons and glial cells is an extension of existing methods based on Granger causality. Granger et al. [59] presented a model-based statistical approach to explore the causality between two variables by examining whether the prediction of a time series of one variable could be improved by incorporating information on the past values of the other [60]. Kim et al. [41] applied Granger causality to functional connectivity analysis of spike sequences; they performed a statistical test based on the log-likelihood of the autoregressive model of spike sequences. Our method presented in the current study can be seen as an extension of Kim et al.'s method that utilized the cross-validated likelihood for model selection. By use of the cross-validated likelihood, we could allow the actual underlying process to be different from the process hypothesized by GLM, while the original Granger causality-based method assumed that they were exactly the same.

Schleiber et al. presented another kind of model-free approach [36] to identify the causality between multiple variables. They utilized transfer entropy, which was used to measure improvements in the prediction of one time series by knowing the past values of another. No distribution of variables needs to be assumed because of the model-free computation of entropy in this approach. One possible drawback in the method of transfer entropy is that it can be difficult to incorporate effects in multiple variables and non-stationarity in the underlying stochastic process due to the lack of direct modeling. In contrast, we can apply our method to non-stationary activities of neurons and glial cells by introducing a time-varying spontaneous firing rate to the likelihood model (Eqs. (1) and (2)).

Our GLM is novel particularly in that it combines a Bernoulli point process model to represent binary neuronal spikes [28], [61] and a vector autoregressive model [62] to represent graded glial activities. The vector autoregressive model has been widely accepted in the field of statistical time-series analysis [63]. Although both these models are known, there have never been any studies in neuroscience that have employed a hybrid stochastic model that could simultaneously deal with both discrete and continuous time-series like those in neuron-glia systems.

### High-throughput of proposed method

The most important advantage of functional connectivity-based approaches is their high throughput. The functional connectivity-based approach enabled us to extract essential structures of the neuron–glia system even from a relatively small amount of data that consisted of 10-min time series of Ca2+ imaging in comparison with their pure anatomical connectivity-based counterparts, like those by electron microscopes [64]. The reasonable performance of our method in artificial networks (85% accuracy from activity time series of 1280 s; Fig. S8; see ‘Validation using artificial data’ section of Text S1) suggests that our identified functional connectivities are biologically and statistically plausible. The functional connections estimated with our method are expected to approach true ones in the network (Fig. S8) as the amount of data increases. If there are many unobservable neurons or glial cells, on the other hand, the meaning of functional connectivity may become ambiguous. However, the advantages of functional connectivity-based approaches will increasingly grow in various neuroscientific scenarios with rapid advances in *in vitro* and *in vivo* imaging techniques and increased access to more widespread and longer measurements. A possible future direction is to explore the fusion of functional connectivity-based methods and anatomical methods. Moreover, the response functions estimated with our method have a meaning on their own; they represent the entirety of synaptic connections that not only include ionic factors but also metabotropic factors.

### Limitations of proposed method

Our functional connectivity analysis was based on an assumption that the Ca2+ activities of cells were independent conditional on their history (see ‘Generative model and MAP estimation’ in Methods). This assumption was equivalent to ignoring neuron–glia interactions whose durations were shorter than the sampling interval (10 ms) in this study. Nevertheless, interactions with such a short time scale can play important roles in neuron–glia networks. An existing study that has proposed the max entropy model, for example, has discussed this possibility [65], [66]. For the following two reasons, however, we believe that our assumption will not negatively affect the reliability of our identification of the interactions with relatively long time scales (between 100 and 500 ms), which is the main target of our functional connectivity analysis.

First, we found that the intensity of our response functions were likely to shrink to 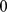 as the delay time approached 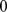 ms (Fig. 6 (left)). This, in particular, means that high frequency responses did not take place around 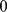 ms. This ruled out the possibility for major interactions on shorter time scales because such interactions most likely triggered high frequency fluctuations in the response functions.

Second, our functional connectivity analysis was based on the difference in cross-validated likelihoods. It would have been unlikely that our abandonment of short term interactions would have severely deformed our computation of cross-validated likelihoods. Even if it had introduced some bias into their evaluations, the bias could be “cancelled out” as we took their differences into account. As such, our method was quite robust against bias that might have resulted from ignoring interactions on smaller time scales. It should be noted that the probability of multiple spikes in 10-ms bins was quite small because the spike frequency (below 1 Hz) in our observation time series was low.

We conducted 10-fold cross-validation (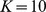) in the time-series analysis. Since we uniformly segmented the whole time series to subseries with a length of 60 s in the cross-validation procedure, interactions with time scales longer than 60 s were simply ignored.

### Bulk numbers of connections in surrogate method

Since the optimal network structure was searched by iterative applications of local searches and hence did not necessarily assess the whole set of identified connections, the bulk number of identified connections was statistically evaluated by means of the surrogate method in which null hypothesis assumed there were in fact no connections in the network (see ‘Surrogate method’section in Methods) [67]. According to the surrogate method, we artificially created time series for neurons and glial cells separately by applying cyclical rotation to the original neuronal time series and phase randomization in the frequency domain to the original glial time series found in the observation dataset. The temporal relationships with other elements in the network were destroyed in the surrogate time series, while preserving important statistical features of its own like those in the distribution and autocorrelation. We then compared the number of connections identified by our method from the actual data against that with the surrogate time series, which led to a statistical evaluation of the bulk number of identified connections.

### Insignificant neuron-to-glia connections

Our functional connectivity analysis was based on iterative applications of local searches for the network structure with the largest cross-validated likelihood. Since multiple hypothesis testing underlies this algorithm, some connections might have been detected by chance even if there had in fact been no connections between neurons and glia. To examine the false positive detection, we used the surrogate method to determine whether the number of identified connections was larger than that found by chance (see ‘Surrogate method’ section in Methods) [67].

We found that the number of identified glia-to-neuron connections was significantly large through surrogate analysis, while that of the neuron-to-glia connections was not. Further, the small and inhibitory neuron-to-glia interactions were inconsistent with the excitatory interactions reported by preceding experimental studies [8], [49]. This inconsistency may be reconciled if we consider the dependence of neuron-glia interactions on the frequency of neuronal firing. Such a frequency-dependent regulation has been discussed within the context of glia-to-neuron connections [19], [43], and a similar regulation might also be realized in neuron-to-glia connections. Note that clear excitatory neuron-to-glia interactions were found through experiments that induced high frequency bursting activities in neurons [8], [49]. On the other hand, the frequency of neuronal activities in our imaging experiment was low (0.03 Hz–1 Hz). Thus, the excitatory neuron-to-glia interactions might have been too weak to have been detected in this low-frequency situation. It is also possible that the Ca2+ active region within the astrocyte's cell body and the sites of neuron-to-glia interactions were so far apart in our imaging experiment, which mostly measured the cell body, that it could not provide us with sufficient information to identify the actual neuron-to-glia connections.

### Neuron-to-glia connections with positivity constraints

Although existing studies have shown that neural spikes cause an increase in glial Ca2+ activity [3], our functional connectivity analysis did not take this known fact into account. The results may change when we assume that all the neuron-to-glia interactions are excitatory. This assumption is equivalent to forcing the response functions, 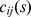, from neurons to glial cells to be positive (see ‘Positivity constraints to response functions from neuron to glia’ section in Methods). We identified nine neuron-to-glia connections out of 288 pairs with the positivity constraints; we found functional connections from neurons to glial cells 2, 4, and 5, but no connections to other glial cells (Fig. S9). When we validated the set of identified connections with the surrogate method, the *p*-value of the number of connections was too large to accept any neuron-to-glia connections. This suggests that, even under the new constraint, neurons do not directly affect glial cells when neurons and glial cells are spontaneously behaving. We compared the cross-validated likelihood between our original model (without the positivity constraints) and the modified model with the positivity constraints on the basis of the distribution of 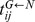. We only considered the set of 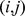
's corresponding to the pair of cells for which our method detected a functional connection. The standard error of the mean (SEM) of these 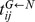 was 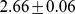 for the original model, and 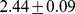 for the modified model. These results indicate that the original model was better than the modified model support our speculation that the model with the positivity constraints did not necessarily capture the nature of the spontaneous *in vitro* activities of neurons and glial cells in the hippocampal CA3 circuit.

## Methods

### *In vitro* Ca2+ imaging

We prepared the hippocampal slice cultures from postnanal, day 7 Wistar/ST rats (SLC). We applied refrigeration anesthesia to the rat pups prior to extracting their brains. We sliced the brains into 300 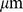 thick slices in aerated, ice cold Gay's balanced salt solution supplemented with 25 mM of glucose. Entorhino-hippocampal stumps including the CA3 region were excised and cultivated on Omnipore membrane filters (JHWP02500, Millipore) placed on plastic O-ring disks. The cultures were fed with 1 ml of 50% minimal essential medium, 25% Hanks' balanced salt solution, 25% horse serum, and antibiotics in a humidified incubator at 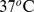 in 5% CO2. They were used for the experiments on days 7 to 14 *in vitro*, and the medium was changed every 3.5 days. We washed the slices three times on the day of the experiment with oxygenated artificial cerebrospinal fluid (aCSF) consisting of (mM) 127 NaCl, 26 NaHCO3, 3.3 KCl, 1.24 KH2PO4, 1.2 MgSO4, 1.2 CaCl2, and 10 glucose and bubbled them with 95% O2 and 5% CO2. The slices were transferred to a 35-mm dish filled with 2 ml of dye solution and incubated for 40 min in a humidified incubator at 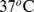 in 5% CO2 with 0.0005% Oregon Green 488 BAPTA-1AM (Invitrogen), 0.01% Pluronic F-127 (Invitrogen), and 0.005% Cremophor EL (Sigma-Aldrich). The slices were then recovered in aCSF for >30 min, mounted in a recording chamber at 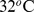, and perfused with aCSF at a rate of 1.5–2.0 ml/min for >15 min. The hippocampal CA3 pyramidal cell layer was imaged at 100 Hz using a Nipkow-disk confocal microscope (CSU-X1, Yokogawa Electric) equipped with a cooled CCD camera (iXonEM+DV897, Andor Technology), and an upright microscope with a water-immersion objective lens (16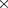, 0.8 numerical aperture, Nikon) [40]. The area we observed is depicted in Fig. 1A. Fluorophores were excited at 488 nm with a laser diode and visualized with a 507-nm long-pass emission filter. We did not see any photodamage during the period of observation; however, we did observe weak photo-bleaching (Figs. 1D and G. Also see [68], [69]). We removed the effect of photo-bleaching by preprocessing the data as described below.

### Pre-processing

We performed the Ca2+ imaging (Fig. 1B) for 10 min (600 s) according to the experimental procedure above. Our imaging yielded a time-lapse image dataset that consisted of 60,000 image frames. The visual field of single image frames was 184 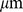 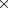
94 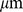 (184
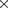
94 pixels). We extracted regions of interest (ROIs) in the first step of image preprocessing, as follows. We applied a spatial smoothing filter (2D Gaussian filter with 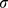 = 1 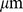) to each image in the time lapse. We calculated the average and standard deviation (SD) of fluorescence signals over the observation period for each pixel along this filtered image series. We then specified the neighborhood (a ball with a radius of 3 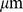) of each local maximum of the average fluorescence intensity as an ROI. We identified a total of 170 ROIs (Fig. 1C). We computed the average signal intensity over the pixels in each of the 170 ROIs, and arranged the average signal intensity along the 60,000 frames that constituted the signal time series of the ROIs (Fig. 1D). We then decomposed the signal time series into a baseline series and activity series on all the ROIs by iteratively applying the following procedure until the baseline series converged. Beginning with the initial baseline series set as flat at the average, we detected all the timepoints inside one SD of the baseline series as inliners, and replaced the baseline series with the new one connecting the inliners. We re-calculated the SD based on the new baseline series in the next application of this procedure. We then dissociated another baseline series. This baseline detection was in essence a detrending procedure; it removed the trends due to possible photo-bleaching. We defined spiking events as peaks of time series with substantially larger intensities than the baseline (with a fixed difference) for each ROI. We detected 48 ROIs out of the 170 ROIs, which indicated sufficient numbers of spiking events (0.03–1 Hz, Fig. 1E). We confirmed these 48 ROIs corresponded to neuronal soma by visually inspecting them. The peaks of the neuronal Ca2+ spikes were found to have similar intensities, and we observed no buildup activities (Fig. 1D). We therefore deemed it safe to interpret each Ca2+ spike with a width of 10 ms to be a single spike. As such, the activity over each of the 48 ROIs was recorded as a binary time series.

We selected six ROIs, other than the 48 neuronal ROIs, as regions representing glial cells, based on their morphologies (by visual inspection) and fluorescence levels. We particularly selected small cells with high fluorescence levels because such cells were likely to be astrocytes [34]. The radius of each ROI was re-set individually to a smaller value than that of the neurons because we only found six glial ROIs. We used the signal average over each glial ROI as the measure of glial activity (Fig. 1G) and arranging it over 60,000 frames constituted the activity time series. We applied individual linear detrending to each glial time series to remove slow trends possibly induced by photo-bleaching. We then applied a temporal Gaussian filter (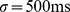) to remove high frequency noise and shot noise. The glial time series thus obtained is depicted in Fig. 1H. We assumed that the activity of astrocytes had a linear relationship in the analysis that followed with the signal intensity measured by Ca2+ imaging.

### Generative model and MAP estimation

Generative modeling was adopted to statistically describe the Ca2+ signals of neurons and glial cells. We introduced a prior distribution to avoid overfitting due to the finite/small size of collected data in the experiments. The model parameters were estimated with the MAP method.

Let 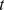 index the image sampling time over the observation, 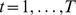; in our particular case, 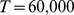. We have activity series of neurons 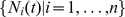 and glial cells 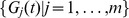 after preprocessing, where 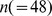 and 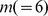 correspond to the numbers of neuronal and glial ROIs. As glial activity is continuous, 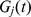 is a series of discrete values sampled from a continuous function of time. 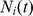 can be seen as a unit point process; 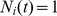 when the *i*-th neuron emits a spike at time 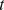, or 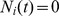 otherwise. Our sampling interval was 10 ms within which every neuron was well assumed to have produced at most one spike in our imaging experiment (see ‘Pre-processing’ section). We normalized the activity time series of the *j*-th glial cell 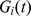 individually, so that its average was zero and variance was one. This normalization was performed because glial cells exhibited different initial fluorescence levels due to variations in light absorption. For simplicity, let 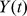 denote the activities of all the elements, 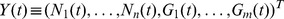, where *^T^* is a transpose. The vector, 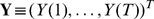, will be called the observation time series after this. We assumed that 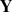 would obey a stationary and conditionally independent Markov chain of order 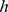, which included an autoregressive process of order 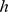 as a special case. When we use the term Markov, our models of interest may include those in which the dependence of the current state on past states is non-linear.

Below, we provide the likelihood of 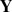, 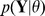 based on our generative model, where 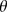 is the parameter vector. Let 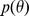 be its prior distribution. Bayes' theorem tells us that the posterior distribution of the parameter vector is given by 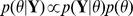. Given an observation time series, 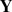, the parameter-vector estimate, 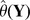, is the 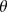 that maximizes the posterior distribution (i.e., the MAP estimation). Our generative model is based on a Markov chain model where the neuronal and glial activities at present are assumed to be mutually independent but dependent on their past activities. More precisely, 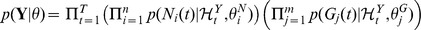, where 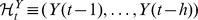 is the history of activities of all the components with a maximum time lag, 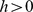. We allowed all neurons to have their own parameters 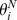 and all glial cells to have their own parameters 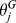. That is, 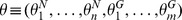. Moreover, the maximum time lag, 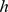, could be differently set for individual types of interactions (see below).

A spike production by the *i*-th neuron with a fixed time interval was assumed to obey a Bernoulli process with logistic regression [32], [70]

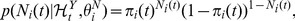
(1a)

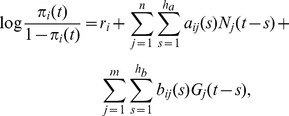
(1b)where 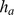 denotes the maximum time lag (history window sizes) from neurons and 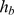 denotes the maximum time lag from glial cells (Fig. 2A). The generative model above is an instance of GLMs, in which the parameter vector of neuron 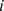 is given by 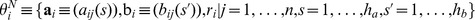, where 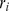 represents the spontaneous firing rate of neuron 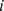, and 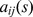 and 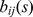 denote the response functions from neuron 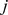 to neuron 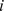 and from glial cell 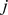 to neuron 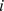, which are defined over the history window sizes 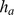 and 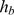, respectively.

The activity of glial cell 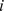 is given by a vector autoregressive (VAR) model disturbed by white Gaussian observation noise, which is another instance of GLMs. More precisely, 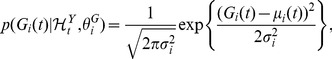
(2a)

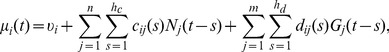
(2b)where 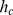 denotes the maximum time lags (history window sizes) from neurons and 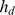 denotes the maximum time lags from glial cells. The parameter vector of glial cell 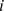 in this VAR model is given by 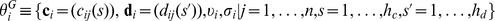, where 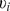 is the bias of glial cell 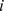 and 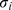 is its variance. Also, 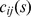 and 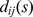 denote the response functions from neuron 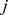 to glial cell 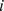 and from glial cell 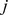 to glial cell 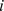, which are defined over the history window sizes 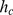 and 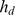, respectively. We have used the notations, 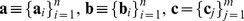 and 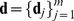, in this paper to represent the sets of response functions between neurons, from glial cells to neurons, from neurons to glial cells, and between glial cells, respectively. The whole GLM for the neuron-glia system above is a state-space model with internal deterministic processes based on a combination of logistic regression and VAR models. The model reduces to a couple of independent GLMs if there are no interactions between the neuronal and glia networks, i.e., 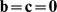.

### Prior distribution

Here, we explain our prior setting of the model parameters in our GLM. We introduced a prior distribution to the parameters representing the response functions, 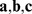 and 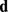, to make the response functions sparse, which is preferred in avoiding overfitting to relatively small datasets, in addition to smoothing with respect to the lag time. Such a prior distribution is given by 
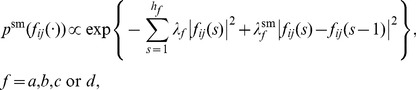
(3)where tuning constant 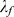 controls the L2-sparseness of the response functions and 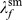 controls their smoothness. We granted independent, noninformative priors 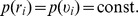 and 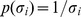 to parameters 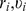 and 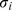 (Eqs. (1) and (2)). In summary, we put 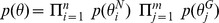, 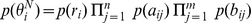, and 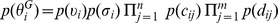. These parameters and their prior distribution are summarized in Table S3.

The prior based on L2-sparseness would be preferable for increasing the cross-validated likelihood of the model [71] by effectively reducing the sensitivity of the model to noise inevitably involved in a relatively small dataset. The smoothness prior would reduce the effective space in which the response functions exist and hence would be beneficial to improve the cross-validated likelihood. Although the time scales of neuron-glia interactions may span a wide range, fluctuating from several tens of milliseconds to several hours [6], [12], [14], our current study focused on specific types of interactions that lasted for several hundreds of milliseconds. Our prior setting that preferred smooth response functions was also considered to work in removing neuron-glia interactions with shorter time scales.

### Efficient estimation of parameters

By applying Bayes' theorem to the likelihood and the prior distribution above, we have the following log posterior 
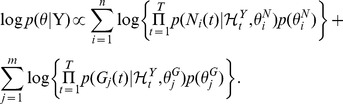
(4)


We obtained the parameter vector, 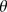, that maximized the log posterior above; the expression above suggests that this MAP estimation can be individually performed for each 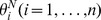 and for each 
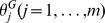
. This individuality also suggests the ability to apply parallel computation to the estimation of parameters.

Fortunately, our set of MAP estimates is unique because our generative model is an instance of GLM [72] and a strictly convex prior distribution also makes the posterior distribution convex. This allows us to use efficient optimization algorithms. When maximizing the first term in Eq. (4) with respect to 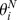, we used a limited-memory Broyden-Fletcher-Goldfarb-Shanno (BFGS) method [73], which is a variation of a quasi-Newton method, to conserve the memory necessary for optimization. The second term in Eq. (4) is a convex quadratic function. We can therefore use a simple linear algebra to estimate 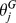.

### Functional connectivity analysis

Our functional connectivity analysis between neurons and glial cells was based on a comparison of the cross-validated likelihood, i.e., the model's reproducibility for the activities in a validation dataset, between two different network structures. If there were two different network structures, one with a certain neuron-glia connection and another without the connection, and the latter demonstrated a larger cross-validated likelihood than the former, then, the connection was not considered to be included in our neuron-glia system. According to the 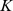-fold cross-validation with 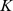 being 10, we partitioned the time series 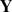 into 10 subseries; we used nine of these subseries to train the model (“training dataset”), and calculated the model-likelihood of the one remaining subseries (“test dataset”) as the cross-validated likelihood of the model. The neuron-wise, test-dataset-wise cross-validated likelihood of the activity of the *i*-th neuron, evaluated on the *k*-th test dataset for a network structure, 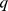, was given by 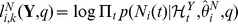, where 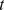 indexes the re-arranged sampling time (sample number) in the *k*-th test dataset, and the parameter vector 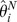 was determined by using the training dataset other than the *k*-th test dataset under network structure 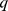. By taking the average of the neuron-wise, test-dataset-wise cross-validated likelihood over the 10 test datasets, we have the neuron-wise cross-validated likelihood of the *i*-th neuron, 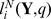. Then, taking the average over all the neurons, we have the cross-validated likelihood of network structure 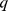 as 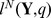.

Similarly, we defined 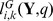 as the glia-wise, test-dataset-wise cross-validated likelihood of the activity of the *i*-th glial cell evaluated on the *k*-th test dataset for network structure 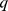. We also defined the *i*-th glia-wise cross-validated likelihood, 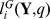, and likewise the cross-validated likelihood of network structure 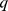 as 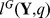.

When evaluating the connections from the *j*-th glial cell to neurons, we compared the cross-validated likelihood between two different network structures, 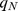 and 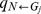, to which different constraints were introduced. The constraint given to 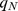 was 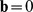, i.e., there were no connections from any glial cell to any neuron. The constraint given to 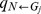 was 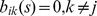 for all 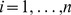, i.e., there were no connections from glial cells to neurons other than from the *j*-th glial cell. We evaluated the neuron-wise cross-validated likelihood, 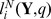, 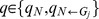, for each of the two network structures after we had estimated their individual model parameters. Observe that 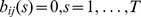 yields 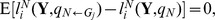 where the expectation is with respect to the GLM (Eq. (1)) with the true parameter vector plugged in. This observation suggests that we can use the difference in the cross-validated likelihood, 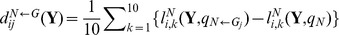, to evaluate the effect from a specific functional connectivity from glial cell 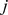 to neuron 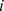, which is represented by the response function, 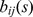.

As it is difficult to obtain the analytical form of the distribution for the stochastic variable, 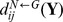, there is no theoretical way to perform a statistical test based on it. To construct a statistical test in a practical manner, therefore, we assumed that the difference in the neuron-wise, test-dataset-wise cross-validated likelihood, 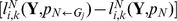, would obey a normal distribution with a zero mean and variance 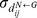, and designed a *t*-statistic: 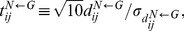
(5)where 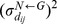 is the unbiased variance of the difference in the cross-validated likelihood, 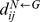, calculated in the cross-validation process. By simply assuming the normality of the stochastic variable, 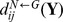, we can make 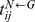 to follow a *t*-distribution. We can then rely on the standard *t*-test, when evaluating each connection from glial cell 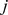 to neuron 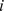. Indeed, this *t*-statistic assumption is not very accurate because the cross-validation samples are not independent of one another and the stochastic variable does not obey a normal distribution. However, the advantages of the *t*-statistic assumption on 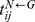 outweigh the disadvantages; we can evaluate the stochastic uncertainty of 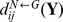 up to the second order moment by using this token.

We took the opposite approach when evaluating connections from neurons to a particular single glial cell, 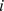. We compared two different network structures in a similar way to that above with a fixed glial cell of interest, i.e., the *i*-th glial cell: the network with no neuronal connections to the glial cell (i.e., 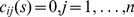), and the network consisting of all possible connections. We defined the mean of differences in the glia-wise, test-dataset-wise cross-validated likelihood of the *i*-th glial cell by 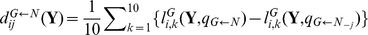.

A *t*-statistic of the difference in the glia-wise cross-validated likelihood was similarly defined as 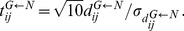
(6)


We treated the connections from glia to neurons differently from those from neurons to glia in this study. The main principle of our search for the optimal network structure was to begin the search from a network structure with the highest cross-validated likelihood possible (see ‘Methods Overview’ section in Results. Some details are also given in Text S1). While the network structure with no neuron-to-glia connections exhibited a higher glia-wise cross-validated likelihood than the network structure with full neuron-to-glia connections (full network), the neuron-wise cross-validated likelihood of the full network was lower than that of the structure with no glia-to-neuron connections (Fig. S1). Also, we resorted to an incremental search algorithm by considering the intractability of a full search over the whole space of all possible network structures. The search algorithm we adopted converges to an optimal network structure if we begin the search from a heuristically chosen structure with a high cross-validated likelihood. The cross-validated likelihood of the network structure monotonically increases and necessarily converges in this search algorithm because we only adopt a new structure when the cross-validated likelihood increases whereas the number of possible network structures is huge but still finite.

### Surrogate method

We explored a statistical test based on the surrogate method to statistically examine the number of detected connections under the null hypothesis of no causal connectivity. We need to construct surrogate neuronal or glial activities that might have been observed under the null hypothesis, only from the observation time series.

In order to evaluate the number of detected connections from the *i*-th glial cell to neurons, we generated the surrogate glial activity (Ca2+ signals) of the *i*-th glial cell (called the original glial cell below) 1000 times based on the Iterated Amplitude Adjusted Fourier Transform (IAAFT) method (for details, see Text S1) [74]. This surrogate glial cell was assumed to have no connections to any neurons in the neuron-glia system, but all other parts of the system remained untouched. Surrogate glial activity in the IAAFT method was generated based on the randomization of phases in the activity time series of the original glial cell. Application of IAAFT to glial activity destroyed the mutual correlation between the original glial cell and all the other network components while preserving the amplitude distribution and the autocorrelation of the activity of the original glial cell (see Fig. S2). We obtained 1000 surrogate datasets by replacing the activity time series of the original glial cell with each of the 1000 surrogate glial activities. We then applied functional connectivity analysis to each of the 1000 surrogate datasets and computed the number of detected connections from the surrogate glial cell to neurons. The empirical distribution constructed from the 1000 surrogate datasets could serve as a null distribution built on the hypothesis that there were no functional connectivities from the original glial cell to neurons. We compared the number of actually detected connections based on the original glial cell's activity against the empirical distribution to compute the *p*-value of the original glial cell's activity.

In the construction of each surrogate neuronal activity, on the other hand, we applied a circular shift to the original neuronal spike time series. This type of implementation is preferable [75] because it can perturb the temporal relationship between neurons, whose activities are surrogated, and other components of the network while preserving its own statistics, such as the distribution of inter-spike intervals, autocorrelation, and self-dependence of the original neuronal activity.

### Tuning parameters

We determined the tuning parameters (tuning constants), 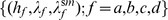 to optimize the cross-validated likelihood by applying heuristic constraints to reduce the space to search for their optimal combination. The parameters to be tuned were maximum time lags (history window sizes) 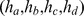 under the heuristic constraints, 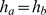 and 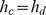, shrinkage parameters of the response functions 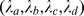 under the heuristic constraints, 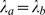 and 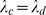, and smoothness parameters of the response functions 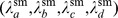 under the heuristic constraints, 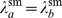 and 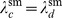. More concretely, we searched discretized candidates 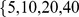, and 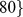 for the best values for both 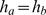 and 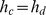, 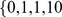, and 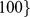 for both 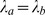 and 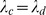, and 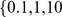, and 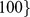 for both 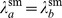 and 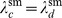, to maximize the cross-validated likelihoods, 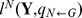 and 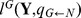. Consequently, we found the optimal values for the tuning parameters were 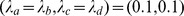, 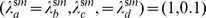, and 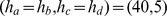.

Here, we applied the heuristic constraints to mainly reduce the search space of the tuning parameters. Such application of constraints is equivalent to having assumed that similar mechanisms govern all receptors on neuronal and glial cells. However, some studies have indicated the possibility that glial receptors might respond differently to neurons and glia [15]. Therefore, we recomputed 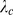 and 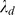 independently (with no constraints) to validate our heuristic constraint 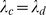, while clumping all the other tuning parameters, and we found that the recomputed parameter values were equal to that with the constraint 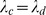. When we carried out the same validation for the constraint, 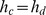, the optimal values without the constraint also yielded the same value as that with the constraint. Further, the overall characteristics of the response functions were found to be fairly robust against the large diversion in the smoothing parameter from its optimal value (Fig. S7).

### Positivity constraints to response functions from neurons to glia

We attempted to introduce a specific constraint, 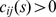 for any 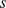, to our GLM, i.e., the connection from neuron 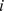 to glial cell 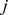 is required to be strictly positive. The parameter optimization (the MAP estimation) of the log posterior with our likelihood and prior distribution is equivalent to the minimization of a specific quadratic cost function. The parameter estimation under the additional constraint, 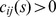 for any 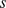, can then be performed by quadratic programming [76], so as to minimize the cost function under the constraint. Based on the 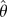 thus computed, we can compute the neuron-wise cross-validated likelihood, 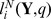 as well as *t*-statistic 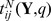 for any network structure 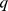. We explained how the introduction of the positivity constraint above affected the results of functional connectivity analysis at the end of the Discussion section.

## Supporting Information

Figure S1
**Model comparison based on cross-validated likelihood.** We compared the models with different assumptions on the network structure, 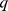 (summarized in Tables S1 and S2), in terms of the cross-validated likelihood of the glial activities, 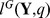 (left panel), and that of the neuronal spikes, 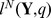 (right panel). Asterisks indicate the presence of statistically significant differences (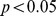, paired Student *t*-test).(EPS)

Figure S2
**Iterative amplitude adjusted Fourier transform (IAAFT) method.** (A) The activity of a single glial cell (glia 2, left panel) and the activity surrogated from the original activity of the same cell by the IAAFT method (right panel). (B) Amplitude distribution and autocorrelation of the original and surrogate glial activities. Note that the amplitude distribution is the same between the original and the surrogate ones (left panels). The autocorrelations are also similar (right panels). (C) The cross correlation between a neuronal activity and a glial activity was destroyed by the IAAFT method. The left (middle) panel shows the phase diagram of the cross correlation between neuron 6 and glial cell 2 (surrogate glial cell 2), in which the abscissa and ordinate denote the activity of glial cell 2 and the spike frequency of neuron 6, respectively. We used bins of 5 s to calculate the spike frequency from the neuronal activity time series. The rightmost panel shows a histogram of the cross correlation coefficient, 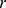, between all pairs of neurons and the surrogate glial cells (the latter are independent of other network components). Note that the histogram spans the range of 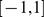 and has a mean of approximately 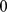. This indicates that the correlation is sufficiently randomized.(EPS)

Figure S3
**Detection of glia-to-neuron connections.** Recall that 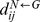 is the gain in the cross-validated likelihood achieved by the addition of the connection from the *j*-th glial cell to the *i*-th neuron, and 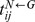 is the *t*-statistics derived from 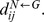 The six panels depict 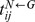 for all pairs of 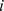 and 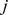. Each panel corresponds to a single glial cell (different index of 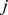) out of the six. We considered a connection from the *j*-th glial cell to the *i*-th neuron significant if the *p*-value of 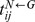 was smaller than 0.05 (marked with an asterisk).(EPS)

Figure S4
**Correlation between pairs of neurons and glial cells between which our method identified connections.** (A) Our method detected a connection from neuron 6 to glial cell 1. Their activities actually exhibited a high correlation (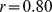, left panel). On the other hand, our method detected no connection from neuron 6 to glial cell 2. Their activities exhibited a lower correlation (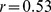, right panel). (B) The histogram of the cross correlation coefficient, 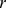, indicates that the magnitude of the cross correlation between the activity pair of a glial cell and a neuron tends to be higher when there is an identified connection from the glial cell to the neuron. This claim could be statistically verified with the Wilcoxon rank sum test (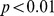). (C) The histogram of the cross correlation coefficient was not significantly different between connected neuron 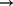 glia pairs and non-connected neuron 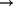 glia pairs (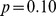, Wilcoxon rank sum test).(EPS)

Figure S5
**Detection of neuron-to-glia connections.** Recall that 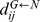 is the loss in the cross-validated likelihood caused by the removal of the connection from the *i*-th neuron to the *j*-th glial cell, and 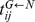 is the *t*-statistics derived from 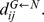 The six panels summarize 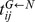 for all pairs of 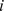 and 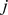. Each panel corresponds to a single glial cell (different index of 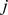) out of the six. We considered a connection from the *i*-th neuron to the *j*-th glial cell significant if the *p*-value of 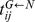 was smaller than 0.05.(EPS)

Figure S6
**Length of identified neuron-to-glia and glia-to-neuron connections.** Boxplots of the length of identified connections. The first, second, third, and fourth columns correspond to the sets of identified connections from glial cells to neurons (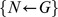), identified connections from neurons to glial cells (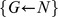), identified connections from neurons to glial cells by applying positivity constraints to the response functions (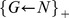), and those in all neuron-glia pairs in the dataset ({Full}). The asterisk indicates that the median length of the identified glia-neuron connections was significantly shorter than those of the bootstrap samples (see Results in the main text) sampled from all the pairs {Full} (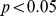).(EPS)

Figure S7
**Robustness against perturbation of smoothing parameters.** The panel on the left indicates the average response functions from glial cells to neurons 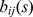 with different smoothing parameter values (red, blue, magenta, and green lines for 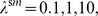 and 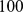, respectively). The panel on the right indicates the average response functions from neurons to glial cells 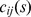 with different smoothing parameter values (red, blue, magenta, and green lines for 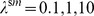, and 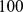, respectively).(EPS)

Figure S8
**Correct rate (accuracy) in reconstruction of glia-to-neuron connections from artificial data.** The error bars indicate the 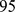
% confidence intervals of accuracy (50 trials).(EPS)

Figure S9
**Detection of neuron-to-glia connections by introducing positivity constraints to their response functions.** We implemented a modified version of our method with positivity constraints on the response functions from neurons to glia. Likewise in the original method, we used the *t*-statistic, 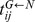, to identify the connections. The modified method identified connections from some neurons to glia 2, 4, and 5 (marked in red). Under this new constraint, we could not detect any connection from neurons to glia 1, 3, and 6. The neurons indexed in green numerals in each panel indicate those with the identified connections to the glial cells marked red, whose removal degraded the cross-validated likelihood of the activity of the red-marked glial cells. The bottom right panel indicates the average for the estimated response functions of the identified neuron-to-glia connections, along with the 95% confidence interval at each delay time.(EPS)

Table S1 **Constraints on network structure for identification of neuron-to-glia connections (Eq. (1)).**
(PDF)

Table S2 **Constraints on network structure for identification of glia-to-neuron connections (Eq. (2)).**
(PDF)

Table S3 **Prior setting of model parameters.**
(PDF)

Table S4 **Tuning parameters and their constraints.**
(PDF)

Text S1 **Supplementary text.** This text file describes the ‘Iterative amplitude adjusted Fourier transform method’, ‘Validation of the proposed method using artificial data’, and ‘Comparison of network structures’.
(PDF)
