# Supplementary material for: A Statistical Method of Identifying Interactions in Neuron–Glia Systems Based on Functional Multicell Ca2+ Imaging
Source: PLoS Comput Biol. 2014 Nov 13;10(11):e1003949. doi: 10.1371/journal.pcbi.1003949 (PMC4230777; doi:10.1371/journal.pcbi.1003949)
Supplement: Table S1 — Constraints on network structure for identification of neuron-to-glia connections (Eq. (1)). (PDF) [file pcbi.1003949.s010.pdf]

Structure	Constraint	Description
$q_{N_{\text{i.d.}}}$	$\mathbf{a} = 0$ and $\mathbf{b} = 0$	Independent neurons
$q_{N_{\text{i.d.}} \leftarrow G}$	$\mathbf{a} = 0$	Neuron-to-glia network, where neurons are independent of one another
q_N	$\mathbf{b} = 0$	Neuronal network
$q_{N \leftarrow G}$	No constraints	Neuron-to-glia network
$q_{N \leftarrow G_j}$	$b_{ik}(s) = 0, \forall k \neq j, \forall i$	Neuron-to-glia network, where only j-th glial cell exists
