# Supplementary material for: A Statistical Method of Identifying Interactions in Neuron–Glia Systems Based on Functional Multicell Ca2+ Imaging
Source: PLoS Comput Biol. 2014 Nov 13;10(11):e1003949. doi: 10.1371/journal.pcbi.1003949 (PMC4230777; doi:10.1371/journal.pcbi.1003949)
Supplement: Table S2 — Constraints on network structure for identification of glia-to-neuron connections (Eq. (2)). (PDF) [file pcbi.1003949.s011.pdf]

Structure	Constraint	Description
$q_{G_{\mathrm{i.d.}}}$	$\mathbf{c} = 0$ and $\mathbf{d} = 0$	Independent glia
$q_{G_{\mathrm{i.d.}} \leftarrow N}$	$\mathbf{d} = 0$	Glia-to-neuron network, where glia are independent of one another
q_G	$\mathbf{c} = 0$	Glial network
$q_{G \leftarrow N}$	No constraints	Glia-to-neuron network
$q_{G \leftarrow N_{-j}}$	$c_{ij}(s) = 0, \forall i$	Glia-to-neuron network without the j-th neuron
