# Supplementary material for: A Statistical Method of Identifying Interactions in Neuron–Glia Systems Based on Functional Multicell Ca2+ Imaging
Source: PLoS Comput Biol. 2014 Nov 13;10(11):e1003949. doi: 10.1371/journal.pcbi.1003949 (PMC4230777; doi:10.1371/journal.pcbi.1003949)
Supplement: Table S3 — Prior setting of model parameters. (PDF) [file pcbi.1003949.s012.pdf]

| Parameter | Prior distribution | Description |
|---|---|---|
| $a_{ij}(s)$ | Smoothness prior Eq. (3) | Response functions from neuron to neuron |
| $b_{ij}(s)$ | Smoothness prior Eq. (3) | Response functions from glia to neuron |
| $c_{ij}(s)$ | Smoothness prior Eq. (3) | Response functions from neuron to glia |
| $d_{ij}(s)$ | Smoothness prior Eq. (3) | Response functions from glia to glia |
| $r_i$ | Uniform prior | Spontaneous activity rate of neuron $i$ |
| $v_i$ | Uniform prior | Bias term of glia $i$ |
| $\sigma_i^2$ | Non-informative prior $\propto 1/\sigma_i$ | Variance of $i$-th glial activity |
