# Supplementary material for: A Statistical Method of Identifying Interactions in Neuron–Glia Systems Based on Functional Multicell Ca2+ Imaging
Source: PLoS Comput Biol. 2014 Nov 13;10(11):e1003949. doi: 10.1371/journal.pcbi.1003949 (PMC4230777; doi:10.1371/journal.pcbi.1003949)
Supplement: Table S4 — Tuning parameters and their constraints. (PDF) [file pcbi.1003949.s013.pdf]

Tuning parameters	Constraints	Description
$\lambda_a, \lambda_b, \lambda_c, \lambda_d$	$\lambda_a = \lambda_b, \lambda_c = \lambda_d$	Shrinkage parameters of $a_{ij}(s), b_{ij}(s), c_{ij}(s), d_{ij}(s)$
$\lambda_a^{\mathrm{sm}}, \lambda_b^{\mathrm{sm}}, \lambda_c^{\mathrm{sm}}, \lambda_d^{\mathrm{sm}}$	$\lambda_a^{\mathrm{sm}} = \lambda_b^{\mathrm{sm}}, \lambda_c^{\mathrm{sm}} = \lambda_d^{\mathrm{sm}}$	Smoothing parameters of $a_{ij}(s), b_{ij}(s), c_{ij}(s), d_{ij}(s)$
h_a, h_b, h_c, h_d	$h_a = h_b,\ h_c = h_d$	History window sizes of $a_{ij}(s), b_{ij}(s), c_{ij}(s), d_{ij}(s)$
