# Supplementary material for: A Statistical Method of Identifying Interactions in Neuron–Glia Systems Based on Functional Multicell Ca2+ Imaging
Source: PLoS Comput Biol. 2014 Nov 13;10(11):e1003949. doi: 10.1371/journal.pcbi.1003949 (PMC4230777; doi:10.1371/journal.pcbi.1003949)
Supplement: Text S1 — Supplementary text. This text file describes the ‘Iterative amplitude adjusted Fourier transform method’, ‘Validation of the proposed method using artificial data’, and ‘Comparison of network structures’. (PDF) [file pcbi.1003949.s014.pdf]

Text S1

A statistical method for identifying interactions in neuron–glia systems based on functional multicell Ca2+ imaging

Ken Nakae[1], Yuji Ikegaya[2,3*], Tomoe Ishikawa[2], Shigeyuki Oba[1], Hidetoshi Urakubo[1], Masanori Koyama[1], Shin Ishii[1,*]

1 Integrated Systems Biology Laboratory, Graduate School of Informatics, Kyoto University, Sakyo-ku, Kyoto, Japan
2 Laboratory of Chemical Pharmacology, Graduate School of Pharmaceutical Sciences, The University of Tokyo, Bunkyo-ku, Tokyo, Japan
3 Center for Information and Neural Networks, Suita City, Osaka, Japan
∗ E-mail:ishii@i.kyoto-u.ac.jp; ikegaya@mol.f.u-tokyo.ac.jp

Contents

1 Iterated amplitude adjusted Fourier transform method **S1**

2 Validation of the proposed method using artificial data **S2**

3 Comparison of network structures **S3**

1 Iterated amplitude adjusted Fourier transform method

In this supplementary section, we describe the Iterated Amplitude Adjusted Fourier Transform (IAAFT) method. This method was adapted for the construction of surrogate glial activities (see 'Surrogate method' section in the main text). The IAAFT is known for its applications in physics [1, 2] as well as econometrics [3, 4]. Described below is the procedure for a single loop of IAAFT. Let $\{G(t)\}_{t=1}^T$ be the original glial time series.

1. Apply the following transformation to $\{G(t)\}_{t=1}^T$.
   (a) Align and rank $\{G(t)\}_{t=1}^T$ by the order of their quantities.
   (b) Generate i.i.d. Gaussian series $\{R(t)\}_{t=1}^T$, align and rank them by the order of their quantities.
   (c) Replace each $G(t)$ with the Gaussian with the same alignment rank.

   Let $\{\mathcal{G}_1(t)\}_{t=1}^T$ be the resulting time series and F_R be its transformation, $\mathcal{G}_1(t) = F_R(G(t))$.
2. Apply the discrete Fourier transformation (DFT) to $\mathcal{G}_1(t)$ and randomize the phases, as follows:
   (a) Apply DFT as $\mathcal{G}_1(t) = \sum_k g(k)e^{i2\pi kt/T}$ and write $g(k) = |g(k)|e^{i\xi_k}$.
   (b) Randomly permute the phases $\{\xi_k\}$. The resulting phases are denoted as $\{\phi_k\}$.
   (c) Obtain $\mathcal{G}_2(t) = \sum_k g'(k)e^{i2\pi kt/T}$, where $g'(k) = |g(k)|e^{i\phi_k}$.
3. Apply the inverse F_R^{-1} to $\mathcal{G}_2(t)$ using the same Gaussian $R(t)$ in the step 1. We call the resulting time series $\mathcal{G}_3(t) = F_R^{-1}(\mathcal{G}_2(t))$.

We repeated these processes until the autocorrelation of $\mathcal{G}_3(t)$ became close enough to that of the original time series $G(t)$. Each surrogate glial activity thus shares the same amplitude distribution as the original glial activity, and its autocorrelation is similar to that of the original. Due to the randomized phases $\{\phi_k\}$ in step 2, on the other hand, the global temporal structure in the surrogate glial activity is completely different from the original. As such, this surrogate procedure destroys the temporal relations between the glial cell and the other network elements while preserving the important statistical characters of the glial activity. In Fig. S2, we plot the amplitude distribution and the autocorrelation of the surrogate glial activities.

We end this subsection with the description of the reason why we surrogated the neuronal activities and the glial activities differently. Recall that we applied a circular shift to neuronal spikes in the surrogate method (see 'Surrogate method' section in the main text). We could not directly apply the same method to the glial activities, because they are, in general, aperiodic. In particular, a forceful application of a circular shift will create a discontinuity at the end of each period, thereby introducing unnecessary outliers which would be harmful for the model estimation. We therefore resorted to the IAAFT in the surrogate method of the glial activities. The IAAFT, on the other hand, is specialized in surrogating the continuous time series like glial activity time series.

2 Validation of the proposed method using artificial data

To further validate the proposed method for identifying neuron–glia networks, we generated an artificial dataset using the generative model (GLM) defined by Eq. (1)(2) in the main text. We then applied our algorithm to the dataset and evaluated the success rate (accuracy) of recovering the simulated functional connections.

We generated 50 sets of artificial neuron–glia networks consisting of $n = 5$ neurons and $m = 3$ glial cells. Each functional connection was randomly added to the network with probability 1/2. For the response functions, we used a form of the alpha function given by $\alpha(s) = s/\tau \exp\{-s/\tau\}$ ($\tau = 30$). The history window sizes were set at $h_a = h_b = h_c = h_d = 10$ (Table S4). Here, the time unit was 10 ms as to correspond to the sampling intervals of 10 ms in our imaging experiment. Also, recall that in our GLM we introduced the constant term r_i that represents the individual spontaneous firing rate. That is, our model is built on an assumption that each neuron fires spontaneously, even in absence of other neurons or glial cells. For our generated model, we set $r_i = 0.3$. Similarly, we set the bias term of the glial activities to be $v_i = 0.3$ and the variance to be $\sigma_i = 0.3$.

On each neuron–glia network, we generated time series of neuronal and glial activities based on iterative applications of Eq. (1) and Eq. (2) as follows. We firstly prepared the initial neuronal and glial activities for the time length $h = 10$. For neurons, we generated the initial spike sequences, $(N_1(t), \ldots, N_n(t))_{t=1}^{h}$, that followed an independent Bernoulli distribution with $p = 0.5$. For glial cells, we generated the initial glial activities, $(G_1(t), \ldots, G_m(t))_{t=1}^{h}$, that followed an independent Gaussian distribution with $(\mu, \sigma) = (0, 1)$. Then, by substituting the series, $(N_1(t), \ldots, N_n(t))_{t=1}^{h}$ and $(G_1(t), \ldots, G_m(t))_{t=1}^{h}$ into Eq. (1b)(2b), we obtained $N_i(h + 1)$ and $G_j(h + 1)$ for $i = 1, \ldots, n$ and $j = 1, \ldots, m$. By repeating this procedure, we obtained $N_i(t)$ and $G_j(t)$ for any i, j and t, i.e., the time series, $(N_1(t), \ldots, N_n(t))_{t=1}^{T}$ and $(G_1(t), \ldots, G_m(t))_{t=1}^{T}$.

We generated the artificial neuron–glia activities over the periods of 10, 20, 40, 80, 160, 320, 640, and 1280 s. For each time period, we simulated 50 time series. We then applied our method to each artificial time series and evaluated the glia-to-neuron connections using $t_{ij}^{N \leftarrow G}$ (Eq. (5)) and the neuron-to-glia connections using $t_{ij}^{G \leftarrow N}$ (Eq. (6)). Fig. S8 shows the average success rate at which our algorithm successfully recovered the artificially prepared connections. We see that the success rate is monotonically increasing with respect to time, and is as high as 85% for 1280 s observation. Most notably, the success rate is already at 70% for 80 s observation. This demonstrates the ability of our algorithm to perform well on relatively short observation time series. The result also suggests that the set of functional

connectivity detected by our algorithm approaches the true connectivity as we gain access to longer and longer observation time series.

3 Comparison of network structures

In our model, we considered four types of functional connections: neuron-to-neuron, glia-to-neuron, neuron-to-glia, and glia-to-glia, represented by $\mathbf{a}, \mathbf{b}, \mathbf{c}$, and $\mathbf{d}$ in Eqs. (1)(2), respectively. Here, we articulate on the contribution of these four types of connections to the cross-validated likelihood. The left panel of Fig. S1 depicts the cross-validated likelihood of the glial activities, $l^G(\mathbf{Y}, q)$, of the four network structures: $q = q_{G_{\text{i.d.}}}, q_{G_{\text{i.d.}} \leftarrow N}, q_G$ and $q_{G \leftarrow N}$. The network structure $q_{G_{\text{i.d.}}}$ assumed $\mathbf{c} = \mathbf{d} = 0$. The network structure $q_{G_{\text{i.d.}} \leftarrow N}$ assumed $\mathbf{d} = 0$. The network structure q_G assumed $\mathbf{c} = 0$. The last one $q_{G \leftarrow N}$ is the full network with no structure constraint. The details of these network structures are summarized in Table S2. Observe that the pair of the network structures q_G and $q_{G \leftarrow N}$ exhibited significantly better cross-validated likelihood for the glial activities in comparison to the pair of the network structures $q_{G_{\text{i.d.}}}$ and $q_{G_{\text{i.d.}} \leftarrow N}$. Note that the first pair is different from the latter pair in that they admit the inter-glia interactions. This suggests that the inter-glial connectivity plays a pivotal role in the overall glial activities. Also, according to the Student t-test, the network structure $q_{G \leftarrow N}$ outperformed the network structure q_G significantly. On the other hand, the difference in the performance between $q_{G_{\text{i.d.}} \leftarrow N}$ and $q_{G_{\text{i.d.}}}$ was statistically insignificant. These observations suggest that neuron-to-glia interactions are necessary in addition to inter-glia interactions to better describe the glial activities.

The right panel of Fig. S1 depicts the cross-validated likelihood of the neural activities, $l^N(\mathbf{Y}, q)$, of the four network structures: $q_{N_{\text{i.d.}}}, q_{N_{\text{i.d.}} \leftarrow G}, q_N$ and $q_{N \leftarrow G}$. The network structure $q_{N_{\text{i.d.}}}$ assumed $\mathbf{a} = \mathbf{b} = 0$. The network structure $q_{N_{\text{i.d.}} \leftarrow G}$ assumed $\mathbf{a} = 0$. The network structure q_N assumed $\mathbf{b} = 0$. The last one $q_{N \leftarrow G}$ is the full model that admits all connections (Table S1). According to the Student t-test, $q_{N_{\text{i.d.}}}$ outperformed $q_{N_{\text{i.d.}} \leftarrow G}$ significantly in the cross-validated likelihood of the neural activities. Similarly, q_N outperformed $q_{N \leftarrow G}$ significantly. In particular, the presence of the full glia-to-neuron connectivity always hurt the cross-validated likelihood. This observation prompted us to hypothesize that the network structure with the best glia-neuron connectivity pattern, which was characterized by the highest cross-validated likelihood, lies near the structure with no glia-to-neuron connectivity. Henceforth, in our quest for the network structure with the highest cross-validated likelihood (see 'Functional connectivity analysis' section in the main text), we began our search from a network structure without glia-to-neuron connectivity.

References

1. Schreiber T, Schmitz A (1996) Improved surrogate data for nonlinearity tests. Phys Rev Lett 77: 635.

2. Schreiber T, Schmitz A (2000) Surrogate time series. Physica D 142: 346–382.

3. Leontitsis A, Vorlow CE (2006) Accounting for outliers and calendar effects in surrogate simulations of stock return sequences. Physica A 368: 522–530.

4. Addo PM, Billio M, Guegan D (2013) Nonlinear dynamics and recurrence plots for detecting financial crisis. N Amer J Econ Finance 26: 416–435.
